# The role of DNA methylation on *Octopus vulgaris* development and their perspectives

**DOI:** 10.3389/fphys.2014.00062

**Published:** 2014-02-24

**Authors:** Eva Díaz-Freije, Camino Gestal, Sheila Castellanos-Martínez, Paloma Morán

**Affiliations:** ^1^Departamento de Bioquímica, Xenética e Inmunoloxía, Facultade de Bioloxía, Universidade de VigoVigo, Spain; ^2^Aquatic Molecular Pathobiology Group, Instituto de Investigaciones Marinas (IIM-CSIC)Vigo, Spain

**Keywords:** DNA methylation, *Octopus vulgaris*, MSAP, paralarvae, aquaculture

## Abstract

DNA methylation is a common regulator of gene expression and development in mammalian and other vertebrate genomes. DNA methylation has been studied so far in a few bivalve mollusk species, finding a wide spectrum of levels. We focused our study in the common octopus, *Octopus vulgaris*, an important organism for neuroscience, physiology and ethology research as well as for human consumption. We aim to confirm the existence of DNA methylation in *O. vulgaris* and ultimately, if methylation plays a role in gene regulation during octopus development. We used a genome-wide approach, methylation-sensitive amplified polymorphism (MSAP), firstly in four different tissues from the same specimens from adult benthonic individuals to test whether gene expression is regulated by methylation. Secondly, we tested the hypothesis that methylation underlies development by assessing MSAP patters from paralarvae to adult developmental stages. Our data indicate that octopus genome is widely methylated since clear differences can be observed, and the methylation pattern changes with the development. The statistical analyses showed significant differences in methylation pattern between paralarvae, where higher internal cytosine methylation is observed, and the three other post-hatching stages. This suggests an important role of cytosine methylation during the first step of development, when major morphological changes take place. However, methylation seems to have little effect on gene expression during the benthonic phase, since no significant effect was revealed in the analyses of molecular variance (AMOVA) performed. Our observations highlight the importance of epigenetic mechanisms in the first developmental steps of the common octopus and opens new perspectives to overcome high mortality rate during paralarvae growth. Thus, better understanding the molecular regulation patterns could lead to new approaches that increase the efficiency of husbandry of this emergent species for aquaculture.

## Introduction

Epigenetic modifications refer to changes in gene expression that occur without altering the underlying DNA sequence (Jaenisch and Bird, 2003; Richards, 2006). These changes are based on a series of molecular processes that can activate, reduce or completely disable the activity of genes (Bossdorf et al., 2008). In fact, these mechanisms are crucial for the regulation of gene expression by allowing cells having the same DNA content to perform different functions (Bird, 2002).

One of the most extensively studied epigenetic modifications in eukaryotic organisms is DNA methylation (Bossdorf et al., 2008). This mechanism consists of a chemical modification of the genomic DNA that involves the addition of a methyl group to a nucleotide, usually the 5'carbon of the cytosine pyrimidine ring, by specific DNA methyltransferases (Mandrioli, 2007; Lu et al., 2008).

Studies of DNA methylation are complicated by naturally occurring variation in methylation among biological entities: different cells, tissues and organisms may show divergent methylation patterns (Yi and Goodisman, 2009). In vertebrates, the genome is heavily methylated mainly in CpG islands of gene promoters, so that methylation is usually involved in gene silencing, development regulation, parental imprinting and X chromosome inactivation. However, invertebrates display a wide range of DNA methylation, from absence in *Caenorhabditis elegans* to low or moderate levels in the case of *Drosophila melanogaster*. Invertebrate methylation is not limited to CpG regions; it can also occur in other DNA coding regions or CpT islands (Colot and Rossignol, 1999; Bird, 2002; Mandrioli, 2004; Su et al., 2011). In fact, methylation in invertebrates seems to be targeted to transcription units (Elango and Yi, 2008). The function and pattern of methylation in invertebrates are unclear, although some studies reveal certain functions such as the involvement of methylation in caste differentiation in *Apis mellifera* (Elango et al., 2009) or the importance of DNA methylation during early stages of development in *Drosophila melanogaster* (Lyko et al., 2000).

There has been little investigation of epigenetics in mollusc, but there is evidence suggesting the presence of CpG methylation in *Mytilus edulis* (Bird and Taggart, 1980), *Donax trunculus* (Petrović et al., 2009) and *Crasostrea gigas*, where DNA methylation has important regulatory functions (Gavery and Roberts, 2010). Furthermore, DNA methylation is crucial to the early development of these species since it takes part in transcription of homeobox orthologs (Riviere et al., 2013).

We have focused our attention in the common octopus, *Octopus vulgaris*. It is a carnivorous cephalopod mollusc, strictly marine that occupies great variety of habitats (e.g., rocks, coral reefs, seagrass beds) in coastal areas worldwide (Vaz-Pires et al., 2004). The commercial worth of *O. vulgaris* is particularly important in Asia and Europe and, it is also important for neuroscience (Grant et al., 2006), physiology (Wells and Smith, 1987), and ethology (Robertson et al., 1994, 2006) research. In addition, the common octopus is a good candidate for aquaculture, since it meets many of the criteria for intensive aquaculture, such as: short life cycle and fast growth, readily adaptive to captivity conditions, high feed efficiency, high reproductive rate, high nutritional value and market price. Unfortunately, the main constraint to achieve an industrial production of *O. vulgaris* relies nowadays in the key to paralarvae rearing, a delicate early stage which suffers almost total mortality under captivity.

As epigenetic changes could play important roles in many development processes that can even be modified by external factors such as temperature, nutrition, light exposure, etc, epigenetic knowledge would be crucial for understanding the development of *O. vulgaris*. Therefore in this work, we attempt a first approach to study epigenetic mechanisms in this species by analyzing the presence and variation of DNA methylation in octopus at different developmental stages.

Rationale of the experiments is as follows. Differences between octopuses at different stages of development were tested using a methylation-sensitive amplified polymorphism (MSAP). It is a modification of the AFLP technique using the same rare cutter *Eco*RI and replacing the frequent cutter *Mse*I with the isoschizomers *Hpa*II and *Msp*I in parallel reactions. Each restriction enzyme recognizes and cleaves the same tetranucleotide sequence 5′-CCGG-3′ but differs in their sensitivity to the methylation state of cytosine (Jeddleoh and Richards, 1996). Therefore, it provides information on the methylation status of the internal cytosine of the CCGG recognition sites. If cytosine methylation has a role in gene regulation or gene expression during development, we should find methylation polymorphism between octopuses at different stages of development. Moreover, if there is a relationship between cytosine methylation and gene expression, different methylation patterns are expected when different tissues of the same individual are compared.

## Materials and methods

### Experimental design

Specimens of *O. vulgaris* were collected by traditional traps, artisanal fishing gear used by local fishermen from the Ría de Vigo (24° 14.09′N 8° 47.18′W), Spain, and transported to the laboratory. Upon arrival, each animal was weighed and measured. Maturity stages were established according to the Hayashi index following Guerra (1975). Eleven immature, 7 maturing, and 17 mature (7 males and 10 females) specimens were selected.

Octopuses were anaesthetized using 7.5% magnesium chloride (Messenger et al., 1985) following ethical procedures (Directive 2010/63/EU). A total of 35 specimens were dissected for the digestive gland, the gills, the hemocytes and the mantle. In addition, paralarvae at hatching (day 1 post-spawning) were obtained from spontaneous spawning of one female octopus, fertilized with sperm of several males, kept in captivity in the aquarium facilities.

### DNA isolation and AFLP genotyping

DNA was extracted from tissue samples composed by eight different pools of four paralarvae each using the NucleoSpin® Tissue Kit BD Biosciences. DNA quality was verified by electrophoresis on 1% agarose gels. After DNA quantification using a Nanodrop 1000 spectrophotometer (Thermo Fisher Scientific), samples were normalized to 100 ng μl^−1^.

The methylation-sensitive amplified polymorphism or MSAP marker was used to detect polymorphism in DNA methylation patterns within tissues of the same specimen and among maturation stages. We assume that widespread alteration in the DNA methylation profile of each sample (specimen and/or tissue) has the potential to drive developmental or physiological variation.

The protocol used for the MSAP technique was adapted from Reyna-López et al. (1997). Briefly, for each sample, 100 ng of DNA was divided into two aliquots to be treated with either *Eco*RI/*Hpa*II or *Eco*RI/*Msp*I. The resulting DNA fragments were ligated with linkers, and then PCR amplified using primers carrying three additional nucleotides. Two primer combinations (*Eco*RI-AAG-*Hpa*II-TCC, *Eco*RI-AAG-*Hpa*II-TAG) were used. *Hpa*II primers were end-labeled using a 6-FAM reporter molecule. PCR products were loaded simultaneously with a GeneScan 500 ROX size standard into an ABI Prism 310 Genetic Analyzer (Applied Biosystems). Fragment analysis and AFLP scoring was performed using GeneMapper v.3.7 software (Applied Biosystems). DNA fragments less than 100 bp in length, longer than 500 bp or less than 70 RFU (Relative Fluorescent Units) were excluded from the analysis due to low levels of reproducibility. Primers and procedures are detailed in Morán and Pérez-Figueroa (2011).

### Data analyses

MSAP profiles, pooled from both primer combinations, were analyzed using the R package msap (Pérez-Figueroa, 2013; http://msap.r-forge.r-project.org). We scored the MSAP fragments as follows: the presence of both *Eco*RI–*Hpa*II and *Eco*RI–*Msp*I products (pattern 1/1) denotes an unmethylated state, the presence of only one of the *Eco*RI–*Hpa*II (1/0) or *Eco*RI–*Msp*I (0/1) products represents methylated states (hemimethylated or internal C methylation) and the absence from both *Eco*RI–*Hpa*II and *Eco*RI–*Msp*I products (0/0) is considered as an uninformative state, as it could be caused by either fragment absence or hypermethylation. Every loci was then classified as either Methylation-susceptible loci (MSL) or Non-methylated loci (NML), depending on whether the observed proportion of methylated states across all samples exceeded a user-defined error rate-based threshold (ERT; 5% by default). Only those fragments showing polymorphism, with at least two occurrences of each state, were used for subsequent analyses (Herrera and Bazaga, 2010).

Analysis in MSAP followed a band-based strategy (Bonin et al., 2007). MSL were used to assess epigenetic variation. The amount of overall epigenetic variation was estimated using the Shannon diversity index (S). Epigenetic differentiation among tissues and maturity stages was assessed by means of principal coordinates analysis (PCoA) followed by analyses of molecular variance (AMOVA; Excoffier et al., 1992). In addition, NML were used following the same strategy to assess genetic variation among maturity stages as their banding pattern depends exclusively on changes of the sequence at the restriction target.

Methylation status was assessed by comparing *Eco*RI–*Hpa*II and *Eco*RI–*Msp*I profiles as standard AFLPs using the option meth (false) implemented in the R package msap.

## Results

### Genome wide methylation analysis

To assess the presence of CpG methylation in *O. vulgaris* banding patterns between *Eco*RI + *Hpa*II and *Eco*RI + *Msp*I were compared. Differences should reflect different states of cytosine methylation at CCGG sites. Considering all the tissues, a total of 462 loci were obtained from adult octopuses, 453 (98%) being polymorphic.

**Figure 1:**
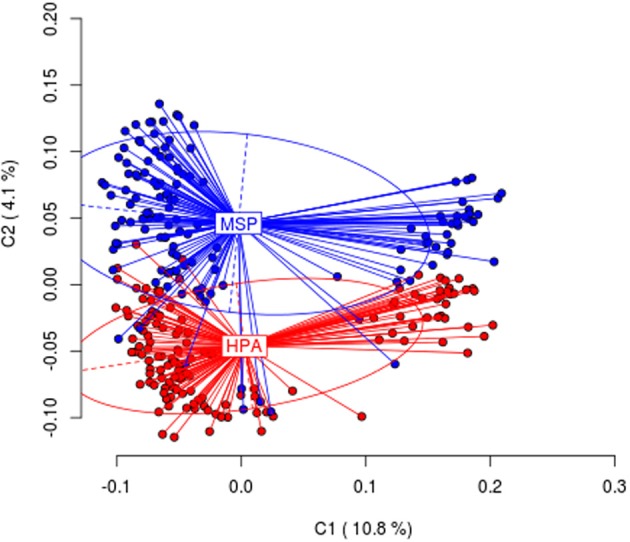
**Results from Principal Coordinates Analysis (PCoA) for the *Hpa*II/*Msp*I comparison in all tissues analyzed**. The first two coordinates (C1 and C2) are displayed with the indication of the percentage of variance explained in brackets. Scores represent individual samples. Labels indicate the centroids of each group. Ellipses represent the dispersion associated to each value. The long axis of the ellipse shows the direction of maximum dispersion and the short axis shows the direction of minimum dispersion.

When all the tissues were considered, differences were highly significant (AMOVA; Φ *ST* = 0.05299, *p* < 0.001). Differences were also statically significant when the four tissues were considered separately (see Table 1). Figure 1 shows the first and second principal coordinates resulting from PCoA which also indicates the degree of differentiation among samples (all tissues included). The second coordinate, accounting for 4.1% of the total variance, showed two clusters, one of them includes samples digested with *Eco*RI + *Hpa*II and the other one includes samples digested *Eco*RI + *Msp*I.

**Table 1:** ***msap* results from *Hpa*II/*Msp*I comparison**.

**TISSUE**	**N° polymorphic loci**	**Variation among*Hpa*II/*Msp*I Φ*ST* (*P*)**
Gill	414 (90% of total)	0.1054 (*P* < 0.0001)
Digestive gland	427 (92% of total)	0.04048 (*P* = 0.0019)
Hemocytes	408 (88% of total)	0.09521 (*P* < 0.0001)
Mantle	396 (86% of total)	0.06963 (*P* = 0.0002)
All tissues	453 (98% of total)	0.05299 (*P* < 0.0001)

*The tissue analyzed, the number and percentage of polymorphic bands and the variation between HpaII and MspI digestions are shown. Brackets indicate the significance value P from the AMOVA test*.

### Methylation patters among different tissues at different developmental stages

To assess differences in cytosine methylation in CCGG sites of different octopus tissues, each tissue (digestive gland, gills, hemocytes and mantle) was considered as a population. Three independent analyses were done, one per each maturation stage. Thus, if the differential gene expression occurring at different tissues is mediated by methylation, we will expect differences among tissues for each maturation stage.

The number of loci classified as MSL by the msap package for immature, maturing and mature was 335, 326, and 336, respectively; whereas the frequency of polymorphic MSL was 68, 56, and 79% accordingly. AMOVA analyses (immature Φ *ST* = −0.01193, *p* = 0.8585, maturing Φ *ST* = −0.02055, *p* = 0.7675 and mature Φ *ST* = 0.05962, *p* = 0.1097) revealed no differences among tissues per each maturation stage. PCoA analyses were not able to differentiate between tissues (data not shown).

### Methylation patters among developmental stages

In order to assess differences in cytosine methylation in CCGG sites along the life cycle, methylation patters of each tissue were compared for immature, maturing and mature octopus. If the differential gene expression occurring during development is mediated by methylation, we will expect differences among maturation stages when methylation patters for a given tissue are compared.

Four independently analyses were done; one for each tissue. The number of loci classified as MSL by the msap package was 321, 348, 325, and 327 gills, digestive gland, hemocytes, and mantle respectively. While the frequency of polymorphic MSL was 67, 71, 71, and 66% for gills, digestive gland, hemocytes and mantle respectively. No differences were found for gills (AMOVA Φ *ST* = −0.01344, *p* = 0.8045), digestive gland (AMOVA Φ *ST* = 0.01411, *p* = 0.114) and hemocytes (AMOVA Φ *ST* = −0.0198, *p* = 0.0807) among maturation stages. However, differences were found in mantle tissue (AMOVA Φ *ST* = −0.03166, *p* = 0.0152). When checking to identify those pairs showing significant differences, the *p*-values were lower than *p* < 0.005 for immature-mature and maturing-mature stages. PCoA analysis failed to show differences between tissues even in mantle (data not shown).

Further analyses were done including paralarvae. Although all the paralarvae share the same mother and therefore are not as representative as the other sets of samples, we think this analysis is potentially informative. Paralarvae banding patterns were compared with immature, maturing and mature octopus banding patterns of mantle tissue. MSAP analysis produced a total of 453 loci, with 359 of them being classified as MSL and 94 of them being classified as NML by the msap package. The frequency of polymorphic MSL was 81% and the frequency of polymorphic NML was 61%. NML analysis reveal no genetic differences between all tested groups (AMOVA; Φ *ST* = 0.0359, *p* = 0.2133), nor genetic differences were found among pairwise analyses. However, the difference in the genome-wide methylation rate between all tested groups was statically significant (AMOVA; Φ *ST* = 0.07304, *p* < 0.0001). Pairwise analyses (Table 2) showed differences between paralarvae and the three other stages.

**Table 2:** **Pairwise AMOVAs between all pairs of the experimental groups**.

	**Inmature**	**Maturing**	**Mature**	**Paralarvae**
Inmature		0.2997	0.0716	**0.0001**
Maturing	0.01028		0.0923	**0.001**
Mature	0.01881	0.0281		**0.0001**
Paralarvae	0.1187	0.1597	0.1421	

*ΦST below the diagonal and p-value above the diagonal. P < 0.05*.

The frequency of the different methylation states (expressed as percentage) in the target sequence (hemimethylation, complete methylation of internal cytosine, full methylation and non-methylated) observed in the different experimental groups is shown in Table 3. Paralarvae showed lower hemimethylation of the target sequence and higher internal cytosine methylation when compared with the other categories.

**Table 3:** **Frecuency (%) of the different states of methylation at the target sequence**.

**Target state (band pattern)**	**Inmature**	**Maturing**	**Mature**	**Paralarvae**
Unmethylated (HPA+/MSP+)	0.2204	0.1950	0.2403	0.2173
Hemimethylated (HPA+/MSP−)	0.1673	0.1894	0.1704	0.1344
Internal cytosine methylation (HPA−/MSP+)	0.1415	0.1326	0.1360	0.1744
Full methylation or absence of target (HPA−/MSP+)	0.4708	0.4830	0.4533	0.4739

**Figure 2:**
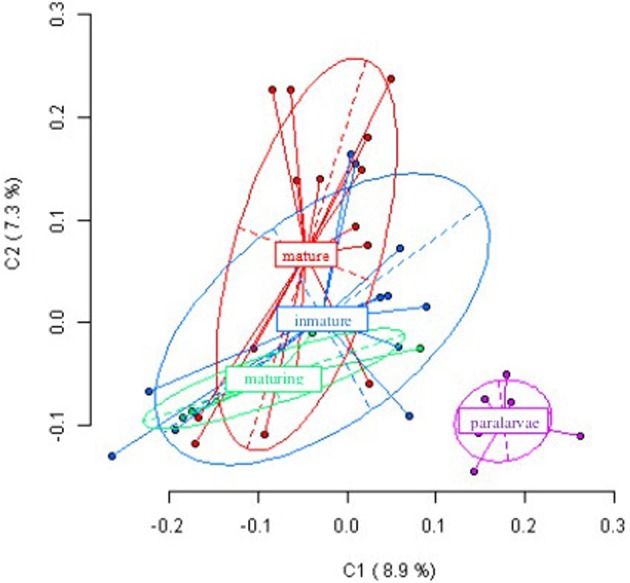
**Results from Principal Coordinates Analysis (PCoA) for the epigenetic differentiation between immature, maturing, adult and paralarvae samples**. The first two coordinates (C1 and C2) are displayed with the indication of the percentage of variance explained in brackets. Scores represent individual samples. Labels indicate the centroids of each group. Ellipses represent the dispersion associated to each value. The long axis of the ellipse shows the direction of maximum dispersion and the short axis shows the direction of minimum dispersion.

Figure 2 shows the first and second principal coordinates resulting from PCoA which indicates the epigenetic variation and the degree of differentiation among the experimental groups. Each dot represents an individual with the exception of paralarvae, where each dot represents a pool of four specimens. The first coordinate, accounting for 8.9% of the total variance, showed two clusters, one of them include paralarvae and the other one the three more developmental stages. Similar results (not showed) were obtained when paralarvae was analyzed considering gill, hemocytes and digestive gland from immature, maturing and mature octopuses.

## Discussion

DNA methylation is a well-known epigenetic process common in a wide range of bacteria, plants, fungi and animals, playing an important role in cell differentiation and gene transcription (Mandrioli, 2007; Su et al., 2011). Methylation in vertebrates occurs through the action of DNA methyltransferases Dnmt1 (maintenance methylation), Dnmt3b (methylation “*de novo*”) and Dnmt2 (unknown function) mainly in the CpGs promoter regions of functional genes (Mandrioli, 2004). In contrast, little is known about methylation in invertebrates. This is mainly due to methylation being absent in the major species of invertebrate model (*Caenorhabditis elegans*) or their levels are too low as in *Drosophila melanogaster* (Yi and Goodisman, 2009). In invertebrates, methylation is known to occur in CpGs as in vertebrates, but also in the CpTs or additional gene promoters (Mandrioli, 2004; Gavery and Roberts, 2010). The only DNA methyltransferase that is present in invertebrates is Dnmt2, whose function is unknown so far (Mandrioli, 2007). Our study in *O. vulgaris* is particularly focused on the methylation of CpG islands. Digestion of DNA samples from *O. vulgaris* with restriction enzymes sensitive to methylation (*Hpa*II and *Msp*I) revealed that the genome of this organism is methylated in the CCGG sequence. However, we cannot rule out the presence of methylation in other parts of genome as CpTs or adenine-rich areas.

DNA methylation appears to be obligatory in vertebrates, therefore functions of DNA methylation have been examined in a large number of organisms for knowing how methylation affects the development of *Xenopus* embryos (Stancheva et al., 2002) or how is it implicated in human carcinogenesis (Jones, 1986; Laird and Jaenisch, 1996). Numerous studies highlight the importance of methylation in development, as in the case of *Mus musculus* (Razin and Shemer, 1995) in which methylation changes are essential for the embryo viability, and in response to environmental conditions (Angers et al., 2010; Feil and Fraga, 2012).

The disparity between vertebrates and invertebrates as well as the small number of invertebrates studied to date, makes the role of methylation in invertebrates not entirely clear. Simmen et al. (1999) suggests that methylated genes of invertebrates must be expressed in place of the silent ones, so that methylation would not be necessary to a viable development, but it is required to increase the expression of genes that, despite being active, could be initiated improperly (Mandrioli and Borsatti, 2006). Bird (1995) however, proposed that DNA methylation in invertebrates may act as a repressor of repetitive sequences and genomic parasites. Jablonka and Lamb (1995) suggest that methylation is related to the development model. Animals with short life cycles and little cell renovation system would use less memory dependent cellular methylation than those with long life cycles and much cell renewal. Regev et al. (1998) argues that these assumptions and functions allocated to DNA methylation are not exclusive and proposes that during evolution, methylation could be recruited for various missions, including genome defense and cellular memory.

The Pacific oyster *Crassostrea gigas*, is the mollusc species where most epigenetics studies have been carried out so far. In this species genetic and epigenetic correlation has been showed in mass selection populations (Jiang et al., 2013). It has been also demonstrated that the involvement of DNA methylation in *C. gigas* gene regulatory functions related to the response to stress and temperature (Gavery and Roberts, 2010). Moreover Riviere et al. (2013) have provided strong evidences for the importance of epigenetic regulation of development in this species.

The results of our study indicate that the genome of *O. vulgaris* is widely methylated, since clear differences can be observed between *Eco*RI–*Hpa*II and *Eco*RI–*Msp*I fragments and cytosine methylation is not tissue specific as it is in vertebrate species (Rodríguez López et al., 2012). MSAP patters between different tissues from the same adult individual are not statistically different suggesting that, in contrast to mammals, genes are not repressed by CG methylation. In addition, we have studied the role of methylation in development by comparing methylation patterns from the same tissues in adult octopus specimens at different maturity stages, finding no differences between them for three of the analyzed tissues: gills, hemocytes and digestive gland. Some differences were observed in mantle when comparing MSAP patterns between early phases of development (immature and maturing) and mature. The probable explanation is the histological and biochemical changes in this tissue associated with reproduction, as suggested by Zamora and Olivares (2004). The octopus muscle acts as an energy reserve of proteins and carbohydrates before and after the spawning, which produces changes in the cellular organization of the tissue. All these transformations may be mediated by cytosine methylation and hence, the differences in the muscular tissue among the different developmental stages. Conversely, striking differences were observed when paralarvae were included in the analysis, showing significant differences in methylation pattern compared to adults. Although result should be interpreted with caution because the paralarvae share the same mother, methylation differences between developmental stages could be appreciated in Figure 2, where each dot represents one individual in adult stages, and a pool of four individuals in the paralarval stage. This suggests the importance of methylation in development from paralarva to adult. This finding although preliminary, is in accordance with those of Riviere et al. (2013) who showed the critical role of methylation during first development steps of *C. gigas*. The common octopus, *O. vulgaris*, has a short life cycle of 12–18 months, rapid growth up to 13% body weight per day and food conversion rates of 15–43% (Navarro and Villanueva, 2003). The newly-hatched octopuses spend their first weeks in the plankton before they settle to a life spent mostly on the bottom. During this phase, they undergo strong morphological changes mainly due to the fast growth of the arms relative to the mantle and number of suckers per arm, but they also undergo changes at physiological and behavior levels (Villanueva and Norman, 2008). In light of these results, it is possible to predict that gene silencing or activation mediated by cytosine methylation is critical during this early period of life when paralarvae are highly vulnerable to food availability and predation, and major morphological changes take place. After this critical period it seems that global methylation has no impact on octopus gene regulation and development, except for the few methylation differences found in the muscle of adults, although specific methylation on particular genes and/or promoters cannot be discarded.

Our study points to an association between methylation and development during a very specific window of time. However, we cannot rule out the presence of other epigenetic mechanisms such as structural modifications of chromatin or small RNA molecules.

Some authors like Rando and Verstrepen (2007) support the idea that patterns of DNA methylation can be altered rapidly in response to environmental changes, integrating these signals in the genome, which would modify the phenotype without changing the sequence of DNA. For example, appropriate dietary bioactive food can modulate gene expression via alterations in DNA methylation (Tammen et al., 2012) because availability of the universal methyl donor SAM has been proved to be essential for healthy development (review by Jiménez-Chillarón et al., 2012). As the main bottleneck in industrial scale cultivation of this cephalopod is the survival of planktonic paralarvae (Vaz-Pires et al., 2004), we think it is worth performing further studies exploring the relationship between paralarvae development and methylation; this would be an interesting line of research, especially for aquaculture purposes.

## Author contributions

Paloma Morán and Camino Gestal conceived and designed the study. Paloma Morán wrote the manuscript and helped with laboratory experiments and statistical analyses; Eva Díaz-Freije performed laboratory experiments and statistical analyses. Sheila Castellanos-Martínez and Camino Gestal performed octopus collection, dissection and assessment of maturity stages. All authors helped with the draft of the manuscript and approved the final document.

### Conflict of interest statement

The authors declare that the research was conducted in the absence of any commercial or financial relationships that could be construed as a potential conflict of interest.
